# The Effects of Insecure Attachment Style on Workplace Deviance: A Moderated Mediation Analysis

**DOI:** 10.3389/fpsyg.2022.813708

**Published:** 2022-04-28

**Authors:** Weijiao Ye, Huijun Zhao, Xiaoxiao Song, Ziqiang Li, Jingxuan Liang

**Affiliations:** ^1^College of Business Administration, Capital University of Economics and Business, Beijing, China; ^2^College of Economics and Management, Huazhong Agricultural University, Wuhan, China; ^3^Anxi College of Tea Science, Fujian Agriculture and Forestry University, Fujian, China

**Keywords:** attachment anxiety, attachment avoidance, organizational deviance behavior, organization-based self-esteem, leader–member exchange

## Abstract

The purpose of this study is to explore why workplace deviance behavior among employees has increased during Corona Virus Disease 2019 (COVID-19) from the perspective of insecure attachment style. Based on attachment theory, we propose and test the effect of insecure attachment style (attachment anxiety, attachment avoidance) on deviance behavior (organizational deviance behavior, interpersonal deviance behavior) *via* organization-based self-esteem using 422 data from Chinese employees. And we further examine the moderating role of leader–member exchange in reducing workplace deviance behavior. The findings show that attachment anxiety and attachment avoidance are both positively related to workplace deviance behavior. Attachment anxiety and attachment avoidance both indirectly predict organizational deviance behavior through organization-based self-esteem. Moreover, leader–member exchange can moderate the indirect effects of both attachment anxiety and attachment avoidance on organizational deviance behavior *via* organization-based self-esteem. This research highlights the fact that employees with insecure attachment style need more care from the organization during the COVID-19 pandemic and demonstrates that one of the key ways in which insecure attachment style increases organization-based self-esteem is by facilitating the development of high-quality leader–member exchange.

## Introduction

Workplace deviation behavior (WDB) is one of the most feared negative behaviors of employees by organizations because it can cause serious harm to the organization, such as deliberately delaying work, wasting resources, stealing and so on (Bennett and Robinson, 2000). These hazards can not only cause huge financial losses to the organization, but also destroy the working atmosphere (Kuhn et al., 2020). The global spread of COVID-19 has increased the unemployment rate, which has also led to an increase in the frequency of employees’ WDB (Liu et al., 2020). Since WDB is harmful and the COVID-19 cannot be eliminated in a short period of time, exploring the reasons for the increase of WDB during the epidemic can help organizations make targeted plans to reduce losses.

Previous studies have explained why employees may exhibit such behaviors from various theoretical perspectives such as interpersonal injustice and abusive supervision (Vogel et al., 2015; Collins and Mossholder, 2017; Akram et al., 2019). However, these studies did not take into account the situation that employees face during the pandemic (Kakarika et al., 2022). Employees bear the risk of contracting the virus at work and worry about losing their jobs due to poor business management, which may increase the insecurity of employees, especially those with more sensitive personalities. For example, (Wagerman et al., 2021) found that anxiety attachment was significantly related to COVID-19 distress, and it was an important predictor of COVID-19 distress, even exceeded the variance of health anxiety, personality and political ideology interpretation. During the pandemic, anxious individuals are particularly vulnerable to poor mental health outcomes and avoidant individuals’ distancing strategies will not be sufficient to buffer against potential effects of the pandemic on mental health outcomes (Vowels et al., 2022). However, existing research overlooked to explore work deviation behavior from the perspective of employee personality under the normalization of the epidemic. So as to fill this gap, we chose the group that is more sensitive to the relationship between the organization and colleagues—the insecure attachment style (IAS).

Existing studies have examined the relationship between COVID-19 epidemics and WDB using a variety of mediating mechanisms, such as emotional exhaustion and job insecurity (Liu et al., 2020; Lin et al., 2021). While these mediating mechanisms explain the increase in WDB during the pandemic, they ignore the role of organization-based self-esteem (OBSE). OBSE refers to the self-worth perceived by employees as members of an organization in the organizational environment (Pierce et al., 1989). It has been found that OBSE can explain employees’ behaviors and outcomes in the workplace. Examples include organizational citizenship behavior, job crafting behaviors, and job performance (Haider et al., 2019; Kim and Beehr, 2021; Liao et al., 2021). Attachment theory believes that infants develop an “internal working mode” of relationships based on caregivers’ responses (Ainsworth and Bowlby, 1991). People with IAS need to be reassured of their worth by the reactions of others (Bowlby, 1969). They also care about their value to the organization, especially in a secure environment (Wei et al., 2007). Therefore, in the context of uncertainty and insecurity caused by COVID-19, OBSE may be the mediating mechanism explaining the increase in WDB caused by IAS.

Previous studies mainly focus on self-control and employee competency uncertainty as moderating factors of WDB (Bordia et al., 2008; Mayer et al., 2012). However, leadership is an important variable in shaping employee attitudes and behaviors in an organization, especially in an environment of increased uncertainty and insecurity (Qian et al., 2020). In the internal working model of attachment theory, it is proposed that individuals with positive external others model are more likely to be close to and self-disclose to the attachment object, while individuals with negative external others model usually adopt inhibitory activation strategy to avoid intimate relationship (Ainsworth and Bowlby, 1991). Scholars have found that leaders are the main attachment objects of employees in an organization (Wu and Parker, 2017), and the internal work model of attachment is a constantly developing structure. Specifically, people may change their perceptions and evaluations of past attachment relationships and integrate new basic beliefs about themselves (Mikulincer et al., 2003). However, existing scholars have not carried out in-depth research on this issue. We therefore suggest that leader–member exchange (LMX) plays a moderating role in the association between IAS and WDB *via* OBSE.

Previous studies have investigated the effect of COVID-19 on WDB (Liu et al., 2020). According to attachment theory, some groups sensitive to changes in organizational environment and interpersonal relationship are more likely to exhibit behaviors that harm the organization after the organization is stimulated by bad environment (Vowels et al., 2022). Given the importance of these groups in predicting WDB during the pandemic, we used attachment theory to investigate the impact of IAS on WDB. This study has three goals: First, testing the influence of the two dimensions of IAS (attachment anxiety; attachment avoidance) on the two dimensions of WDB (organizational deviation behavior; interpersonal deviant behavior). Second, using attachment theory, we study the mediating effect of OBSE. Third, we explore the moderating role of LMX between IAS and WDB. The theoretical contribution of this study has at least three aspects. First, we use attachment theory as our theoretical basis and confirms the impact of IAS on WDB during the COVID-19 pandemic, which can help us examine employees’ WDB from another theoretical perspective. Second, drawing on attachment theory, our study sheds light on why employees with IAS increase WDB in COVID-19, offering a unique lens to uncover the psychological and behavioral impact of this ongoing global crisis on employees. Third, this study use LMX as a moderating variable to explain the important role of leadership for employees who are more sensitive to insecure environments during COVID-19.

## Theoretical Background and Hypotheses

### Workplace Deviance Behavior

The concept of WDB was developed by Bennett and Robinson (2000), who defined it as a voluntary act in which an employee violates organizational norms, threatens the interests of the organization or its members, or both. According to the object of the action, WDB can be further divided into interpersonal deviance behavior (WDB-I) and organizational deviance behavior (WDB-O). Many studies have been performed to investigate the antecedents of WDB (Pletzer et al., 2019; Loi et al., 2020; Wu et al., 2020; Lin et al., 2021). In our study, the influencing factors of WDB were divided into external factors and internal factors. The primary impact of external factors is organizational. For example, interpersonal injustice has a positive impact on WDB by reducing job satisfaction, and organizational power stimulates WDB by making employees feel more frustrated (Lawrence and Robinson, 2007; Collins and Mossholder, 2017). Second, the behavior of employees has always been considered in the context of leaders. Studies have found that abusive supervision has a positive effect on WDB (Mitchell and Ambrose, 2007; Lian et al., 2014; Vogel et al., 2015). Akram et al. (2021) revealed the significant negative influence of abusive supervision on employee creativity in the presence of perceived psychological distress. Leader mistreatment causes WDB through employee hostility (Mayer et al., 2012). Finally, colleagues also have a stimulating effect on employees’ WDB. Workplace ostracism has a positive effect on WDB through the state of self-esteem (Peng and Zeng, 2017).

Internal factors mainly include the personality, cognition and emotions of employees. Scholars have found that Big Five (B5) personality type has a significant predictive effect on WDB (Kluemper et al., 2015; Pletzer et al., 2019). On this basis, using a more complete HEXACO personality test (including honesty, emotionality, extraversion, agreeableness, conscientiousness, openness to experience), Pletzer et al. (2019) found that HEXACO is more accurate than B5 in predicting WDB and thus provides a reference for organizations selecting employees. Insecure attachment styles are associated with WDB due to lower work vigor (Little et al., 2011). In addition, emotional management ability, job insecurity, psychological contract breach, and motivational traits were strong predictors of WDB (Diefendorff and Mehta, 2007; Bordia et al., 2008; Kluemper et al., 2011; Huang et al., 2017). Combined with the current epidemic situation, some studies have found that COVID-19 event strength has a positive impact on WDB (Liu et al., 2020; Lin et al., 2021). The details are shown in Table 1.

**Table 1:** Predictors of workplace deviance behavior.

**Organizational factors**
Organizational power (Lawrence and Robinson, 2007)
Interpersonal Injustice (Ferris et al., 2009; Holtz and Harold, 2010; Collins and Mossholder, 2017)
Workgroup Climates (Bollmann and Krings, 2016)
COVID-19 event strength (Liu et al., 2020; Lin et al., 2021)
**Leader factors**
Leader mistreatment (Mayer et al., 2012)
Abusive supervision (Mitchell and Ambrose, 2007; Lian et al., 2014; Vogel et al., 2015; Akram et al., 2021)
Supervisor-subordinate guanxi (Wu et al., 2020)
**Colleagues factors**
Workplace ostracism (Peng and Zeng, 2017)
Gossip (Zhu et al., 2021)
**Personal factors**
B5 (Kluemper et al., 2015; Pletzer et al., 2019)
HEXACO (Pletzer et al., 2019, 2020)
Emotion management ability (Kluemper et al., 2011)
Employee dissimilarity (e.g., agreeableness dissimilarity, extraversion dissimilarity; Liao et al., 2004)
Personality (Colbert et al., 2004; Mount et al., 2006)
Turnover intentions (Mai et al., 2016)
Job insecurity (Huang et al., 2017)
Psychological contract breach (Bordia et al., 2008)
Motivational Traits (e.g., personal mastery, competitive excellence; Diefendorff and Mehta, 2007)
Attachment style (Little et al., 2011)
Emotional exhaustion (Enwereuzor et al., 2017)

Such studies have not combined environmental factors with the internal factors and have ignored the adverse behaviors of certain sensitive employees in a particularly uncomfortable environment. Unlike these studies, we use attachment theory as our theoretical basis, which can help us examine employees’ WDB from another theoretical perspective. Second, we chose IAS as the predictive variable of WDB because previous studies have found that people with these styles are more sensitive to interpersonal interactions in insecure environments. Simultaneously, we chose to conduct our research in the context of COVID-19 because it allows organizations to gain a clearer understanding of why employees in an environment of insecurity and uncertainty behave in ways that harm the organization. This helps organizations reduce WDB in other insecure and uncertain environments (e.g., leadership change, organizational reform, economic recession). Our study may be more meaningful because factors that trigger employee insecurity during an epidemic may be more obvious and easily documented.

### Insecure Attachment Style and Workplace Deviance Behavior

Attachment theory was developed by Ainsworth and Bowlby (1991), who observed the interactions between infants and their caregivers in their early ages. Attachment theory claims that infants will a positive (or negative) conception of themselves and others based on the responses of caregivers and the consistency of caregivers’ attitudes (Bowlby, 1982). These conceptions become fixed and are maintained as stable attachment styles in adulthood (Richards and Schat, 2011). Based on this theory, Ainsworth and Bowlby (1991) divided individual attachment styles into secure attachment and insecure attachment. Among these types, secure attachment features a positive self-evaluation and positive evaluations of others. Conversely, insecure attachment can be divided into attachment anxiety (negative self-evaluation and positive other-evaluation) and attachment avoidance (positive self-evaluation and negative other-evaluation). Specifically, employees with secure attachment style show higher trust to others (Riggs et al., 2002), while employees with IAS exhibit more anxiety regarding work performance and working relationships and engage in more conflicts with colleagues (Hardy and Barkham, 1994).

Attachment anxiety has negative evaluations of themselves and positive evaluations of others, which makes them more concerned about how the organization and colleagues think of them at work (Jiang et al., 2019). In order to get a high evaluation in the organization and colleagues, when there is a difference of opinion with the organization or colleagues, they will keep silent. Researchers have found that individuals with attachment anxiety show greater stress at work (Harms, 2011) and rarely provide instrumental assistance to their colleagues (River et al., 2019). The uncertainty and insecurity brought about by the epidemic make them feel more psychological pressure (Vowels et al., 2022), and they will be more sensitive to the relationship between themselves and their organizations or colleagues. For fear of being laid off, they pay attention to what the organization thinks of them and form their own judgment. Once the organization displays a bad attitude, it may trigger their organizational deviant behavior (Scrima et al., 2017). Attachment anxiety fulfills all sorts of demands from colleagues at work because they see themselves as bad and others as trustworthy. This is because the demands of infancy are unresponsive and unmet by caregivers, leading them to view themselves as unworthy of love (Ainsworth and Bowlby, 1991). Therefore, in the tense situation of the epidemic, people with attachment anxiety are also more sensitive to interpersonal relationships and more likely to engage in interpersonal deviant behavior than other employees. The study also confirmed that attachment anxiety is positively correlated with job burnout and negatively correlated with job performance (Virga et al., 2019).

Attachment avoidance has positive self-cognition and negative cognition of others, which makes them refuse to communicate with others in the organization, only believe in themselves, and lack team spirit (Schusterschitz et al., 2018). Individuals with attachment avoidance are less likely to participate in organizational citizenship behavior and are more likely to have the intention to leave (Harms, 2011). Therefore, in the coronavirus pandemic with increased uncertainty and insecurity, on the one hand, they may suspect that they will be fired because of their distrust, which may lead to organizational deviation. On the other hand, the suspicion of colleagues suing for personal gain will lead to their dismissal, which may lead to interpersonal deviance behavior. For these reasons, we believe that in the process of workplace interaction with the organization and colleagues, employees with IAS are more likely to attempt to please others because of attachment anxiety, and they tend to avoid communication with their colleagues because of attachment avoidance, which affects their experiences in the organization and can further lead to WDB on their part. This situation became more prominent during the epidemic. Based on the arguments outlined above, we propose the following hypothesis:

*Hypothesis 1*: Insecure attachment style is positively related to workplace deviance behavior.

### The Mediating Role of Organization-Based Self-Esteem

The concept of organization-based self-esteem refers to individuals’ perceptions of their self-worth as members of the organizational environment (Pierce et al., 1989). According to attachment theory, employees with attachment anxiety believe that they are worthless and not worthy of being loved, while others are trustworthy and dependent (Ainsworth and Bowlby, 1991). Thus, these individuals may be more likely to lose support because they tend to seek excessive support at work, which causes problems for the organization and their colleagues. In addition, such individuals are more likely to lose autonomy due to overdependence on others and to reduce their sense of self-worth at work. Mikulincer and Sheffi (2000) proposed that attachment anxiety tends to overemphasize individuals’ perceptions of sadness and overactive negative emotions, thoughts and memories.

Employees with attachment avoidance avoid relying on others in the workplace due to the belief that others will be unavailable when needed, and they avoid intimacy while pursuing autonomy and control (Yip et al., 2018). Accordingly, they rarely ask for help because they are afraid of being rejected, which reduces their sense of presence in the organization. Second, due to this lack of communication and cooperation with the organization or colleagues, employees with attachment avoidance have low self-evaluation of their work performance and believe that they will receive lower performance evaluation from colleagues (Richards and Schat, 2011). Mikulincer and Sheffi (2000) believed that individuals with attachment avoidance resist emotional involvement by suppressing negative and dependence mechanisms, which leads to a negative attitude toward exploration and cognition.

The decrease in self-esteem may automatically activate self-protection measures and promote a fight-or-flight response (McNaughton and Gray, 2000). Thus, we believe that WDB is related to employees’ OBSE. First, employees with low OBSE believe that their role in the organization is worthless and meaningless. These employees engage in behaviors detrimental to the organization in order to get the organization’s attention. Samnani et al. (2014) also confirmed that people are more inclined to engage in counterproductive behavior when experiencing negative emotions. And they thought that they might be looked down upon by their colleagues, so they were more likely to engage in interpersonal deviant behavior because of anger at being looked down upon. Second, according to the self-consistency theory proposed by Lecky (1935), to maintain cognitive consistency between attitude and behavior, individuals take actions consistent with their overall views. OBSE led to greater organizational citizenship behaviors and fewer deviant behaviors (Kim and Beehr, 2018). Numerous studies have supported the mediating role of OBSE between employees’ cognition or work environment and their attitudes and behaviors. These connections include workplace ostracism and organizational citizenship behavior (Li et al., 2021), maternal support and deviance (Liu et al., 2018), abusive supervision and workplace deviance (Vogel et al., 2015), etc. In the process of interacting with supervisors and colleagues, employees with IAS have different experiences in the organization due to differences in information processing methods and self-regulation strategies. These experiences gradually shape their self-esteem in the organization, which in turn affect their behavior. This situation became more prominent during the epidemic. For example, individuals with IAS believe that the epidemic makes their work environment worse and doubles their value in the organization (Vowels et al., 2022). Such a negative perception may further lead to WDB. In the novel context of the COVID-19 and its effects on possible unemployment, employees must pay more attention to their importance in the organization in case they are dismissed. Employees with IAS are more sensitive to their place in the organization or to their coworkers. On the basis of these discussion, we propose the following hypothesis:

*Hypothesis 2*: Organization-based self-esteem mediates the relationship between insecure attachment style and workplace deviance behavior.

### The Moderating Role of Leader–Member Exchange

Leader–member exchange (LMX) emphasizes the establishment of a dual relationship between leaders and followers of mutual respect, trust, and obligation. However, due to time and resource constraints, the relationships between leaders and different members have different characteristics (Graen and Uhlbien, 1995a,b). Specifically, when leaders and followers develop and maintain relationships that feature high-quality social communication, an effective leadership process takes effect. High-quality LMX can develop mutual trust between leaders and employees due to a higher frequency of interactions, and employees’ sense of responsibility and work enthusiasm increases accordingly, which in turn has a positive impact on leaders, employees and organizations (Graen and Uhlbien, 1995a,b). However, low-quality LMX only involves an exchange of resources required to complete basic tasks (Liden and Graen, 1980). In recent years, scholars engaging in LMX research have focused on the expansion of binary relationships at the relationship network and organizational levels (e.g., the role of LMX in a working group’s special transaction performance relationship; Anand et al., 2017). The latest research has also begun to focus on the relationship between LMX and employees’ emotions and behaviors (e.g., organizational citizenship behavior, the emotional tone of employees; Anand et al., 2017; Gooty et al., 2019).

**Figure 1:**
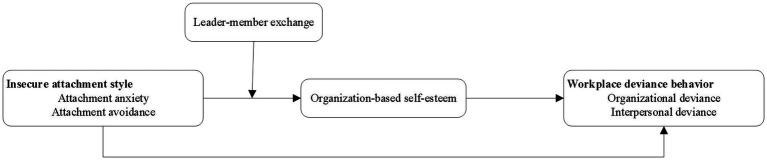
The conceptual model of this study.

Attachment style is developed from the internal working model posited by attachment theory and emphasizes the questions of whether the caregiver responds to the infant’s needs and whether the consistency of that response affects the infant’s internal-self model. Specifically, the caregiver’s response affects the individual’s self-evaluation, that is, the individual’s position concerning whether he or she is worthy of being loved. In turn, employees with IAS are more sensitive to relationships with attachment objects in the organization. According to the research by Bowlby (1982), when persons are physically or psychologically threatened, the attachment behavior system is activated, and they focus on meeting their attachment needs by seeking support from others (Mikulincer et al., 2003). Research concerning adult groups has shown that attachment behavior systems can also be activated in specific interpersonal contexts (e.g., when accepting affirmations, Lee and Thompson, 2011). In fact, the leader plays the role of caregiver to employees in the organization (Wu and Parker, 2017). Shapiro et al. (2016) found that leader turnover is positively correlated with turnover rate and thoughts regarding turnover among subordinates and that it reduces the trust and security of subordinates in the organization. LMX is actually a signal to employees that their work contributions are valued and recognized (Liu et al., 2013). Employees with an attachment anxiety desire the trust and support of their attachment object. Therefore, in the context of high-quality LMX, they feel more valued in the organization. Employees with an attachment avoidance are actually eager to be cared for, although they avoid and reject intimacy with others. They suppress their needs because of fear of rejection. Therefore, high-quality LMX increases the self-worth of employees with attachment avoidance in the organization. Based on the above analysis, we believe that LMX can moderate the relationship between IAS and OBSE and that OBSE plays a mediating role between IAS and WDB. Therefore, it can be further inferred that LMX moderates the indirect effect of IAS on WDB *via* OBSE. In other words, the indirect effect of IAS on WDB *via* OBSE is weaker when LMX is higher rather than lower. Based on these considerations, the following hypothesis is proposed. The research framework of this paper is shown in Figure 1.

*Hypothesis 3*: LMX moderates the indirect effects of insecure attachment style on workplace deviance behavior via organization-based self-esteem such that the indirect effects are weaker when LMX is higher (vs. lower).

## Materials and Methods

### Sample and Procedures

The data used in this study were collected from employees in different industries in the southern, central and northern regions of China (including blue-collar workers and white-collar workers) and used to determine whether our research findings can be extended to employees with different jobs and different income levels. We use two ways to collect questionnaires: first, we use snowball sampling to collect questionnaires from employees around us and ask them to forward the questionnaires to colleagues to fill in. Second, send questionnaires to Chinese companies using the email addresses and phone numbers they publish on Tianyancha app and implore them to pass them on to their employees to fill out. They were randomly awarded between $0.15 and $1.46 after completing the questionnaire. Snowball sampling is widely used in data acquisition in social sciences. Although many studies have found that snowball samples will lead to data bias, the main reason is that sample collection is easy to gather in similar groups, which makes the data biased. However, we did not adopt a single snowball sampling method according to the data acquisition suggestions put forward by Marcus et al. (2017). Because the snowball method produces samples that are closely related, the data we obtain can be fully representative of the people whom we study (employees). However, the snowballing method may lead to high similarity among research objects. To avoid this problem, we collect our data in two ways. We randomly sent short messages and e-mails to companies according to certain parameters and requested that these questionnaires be forwarded to their employees. To reduce common method deviation, we collected the data over two periods and asked respondents to fill in the last four digits of their mobile phone numbers at the beginning of the questionnaire so that the data could be matched later. We clearly informed participants at the beginning of the questionnaire concerning the study method and also told them that the questionnaire was anonymous, that there were no right or wrong answers to our questions, that the survey results would be used only for scientific research, and that they could terminate or withdraw from the survey at any time. Our data collection time was from 04-2020-05. Respondents were asked to test their attachment style, LMX and personal background information at Time-1, and 2 weeks later (Time-2), they were asked to assess their OBSE and interpersonal and organizational deviance behavior. In addition, a self-report method was used to measure WDB because many deviations (e.g., taking property without permission, wasting time at work) are difficult to observe.

At Time 1, we distributed the online questionnaire to 50 employees from the northern, central and southern regions of China and asked those employees to pass the questionnaire on to their colleagues. At the same time, 400 questionnaires were sent in the form of short messages and emails obtained *via* public information such as mobile phone numbers and e-mail addresses registered by the company in the Tianyancha app. We sent emails containing questionnaires to enterprises listed in the app through the official email provided by our school, which could cause enterprises to trust us more; enterprises could freely choose whether to reply to our emails. At Time-1, we collected a total of 503 questionnaires, of which 356 were obtained by snowball sampling and 147 were obtained by sending questionnaire information to enterprise email and short messages. At Time-2, we used the same method as that employed at Time-1 and issued the questionnaires. At this stage, we obtained a total of 453 questionnaires. And we screened the sample data according to the completeness and quality of the questionnaire content and matched the data with mobile phone numbers. Thirty-one invalid questionnaires were excluded. Subsequently, 422 valid questionnaires were obtained, of which 308 were obtained by snowball sampling and 114 were obtained by sending questionnaire information to enterprises’ email and short messages. Taking the questionnaire collected in the first stage as reference, the response rate of the questionnaire was 83.9%. Among participants, 253 (60 percent) were male. Twenty-nine (6.8 percent) of them were under the age of 26 years, 93 (22 percent) were between 20 and 30 years, 123 (29.2 percent) were between 31 and 40 years, 107 (25.4 percent) were between 41 and 50 years, and 69 (16.3 percent) were above 50 years. Thirty-five (8.3 percent) of these participants had master’s degree or above, 126 (29.9 percent) had a bachelor’s degree, 122 (28.9 percent) had an associate degree, 102 (24.2 percent) had a high school/secondary school degree, and 36 (8.5 percent) had a junior high school degrees or below. Seventy-nine (18.7 percent) have worked for 2 years, 95 (22.5 percent) for 3–4 years, 113 (26.8 percent) for 5–6 years, 94 (22.3 percent) for 7–8 years and 40 (9.5 percent) for more than 9 years. 122 (28.9 percent) of them were managers. 111 (26.3 percent) of them have worked with their current leaders for less than 2 years, 129 (30.6 percent) for 3–4 years, 86 (20.4 percent) for 5–6 years, 68 (16.1 percent) for 7–8 years and 27 (6.4 percent) for more than 9 years.

### Measures

To ensure reliability and validity, we used authoritative maturity scales and appropriate translation and back-translation procedures (Brislin, 1979). Furthermore, to ensure that all measurement questions were suitable for our research background, all items were pretested in a group of ordinary employees. All items were measured using a seven-point Likert scale ranging from 1 (strongly disagree) to 7 (strongly agree). All measurement items can be viewed in the Appendix sections (see Appendix).

#### Insecure Attachment Style

We measured attachment anxiety and attachment avoidance with items adapted from the short version of the Adult Attachment Scale ECR-S developed by Wei et al. (2007), which includes six items pertaining to attachment anxiety and six items regarding attachment avoidance. A sample item concerning attachment anxiety was as follows: “My desire to be very close sometimes scares people away.” A sample item pertaining to attachment avoidance was as follows: “I want to get close to my partner, but I keep pulling back.”

#### Workplace Deviance Behavior

We used a scale developed by Bennett and Robinson (2000) to measure organizational deviance behavior and interpersonal deviance behavior. The scale includes seven interpersonal deviance behavior (WDB-I) measures and 12 organizational deviance behavior (WDB-O) measures. A sample item regarding organizational deviance behavior was as follows: “Arrived late to work without permission.” Cronbach’s alpha was 0.96. A sample item concerning interpersonal deviance behavior was as follows: “Made fun of someone at work.”

#### Organization-Based Self-Esteem

We used the 10-item scale developed by Pierce et al. (1989) to measure OBSE. Sample items were as follows: “I matter here” and “I am valuable.”

#### Leader–Member Exchange

We measured employee leader–member exchange with a 7-item scale developed by Graen and Uhlbien (1995a,b). A sample item was as follows: “How much does your leader recognize your potential?”

#### Control Variables

According to the control variable guide developed by Bernerth and Aguinis (2016), we took gender, age, education, job tenure, position, and tenure with supervisor, which are theoretically related to OBSE and WDB, as control variables. Gender was coded as follows: 1 = female, 2 = male. The age coding was as follows: 1 = below 20 years old, 2 = 20–30 years old, 3 = 31–40 years old, 4 = 41–50 years old, and 5 = over 50 years old. The coding of education was as follows: 1 = Master’s degree or above, 2 = Bachelor’s degree, 3 = Associate degree, 4 = High school/secondary school degree, 5 = Junior high school or below. The coding of tenure was as follows: 1 = within 2 years, 2 = 3–4 years, 3 = 5–6 years, 4 = 7–8 years, 5 = 9 years or above. The position coding was as follows: 0 = non-manager, 1 = manager. The tenure with supervisor was coded as follows: 1 = within 2 years, 2 = 3–4 years, 3 = 5–6 years, 4 = 7–8 years, 5 = 9 years and above.

## Results

### Descriptive Statistics and Validity Testing

The averages, standard deviations and correlations of the variables involved in this study are shown in Table 2. The results showed that attachment anxiety (*b* = 0.13, *p* < 0.01) and attachment avoidance (*b* = 0.17, *p* < 0.01) were both positively correlated with organizational deviance behavior. Attachment avoidance was positively correlated with interpersonal deviance behavior. These results provided preliminary support for subsequent hypothesis testing.

**Table 2:** Means, standard deviations, and correlations of all variables in the study.

Variables	Mean	SD	1	2	3	4	5	6	7	8	9	10	11	12
1. Gender	1.60	0.49												
2. Age	3.22	1.16	−0.13^**^											
3. Education	2.95	1.1	−0.01	−0.03										
4. Tenure	2.81	1.24	−0.01	−0.1	−0.01									
5. Position	1.28	0.45	−0.06	−0.09	−0.03	−0.01								
6. Tenure with supervisor	2.44	1.22	0.01	−0.02	0.03	0.02	0.03							
7. Attachment anxiety	4.50	1.63	0.06	−0.01	−0.01	0.04	−0.08	−0.04	(0.86)					
8. Attachment avoidance	4.33	1.73	−0.04	0.02	−0.08	−0.02	−0.05	0.12^*^	−0.06	(0.88)				
9. LMX	3.82	1.76	0.01	0.01	0.05	−0.03	−0.05	0.04	0.01	−0.08	(0.87)			
10. OBSE	3.70	1.76	−0.04	−0.16^**^	0.04	0.25^**^	0.21^**^	−0.03	−0.16^**^	−0.13^**^	0.01	(0.82)		
11. WDB-O	4.42	1.77	−0.05	0.23^**^	−0.32^**^	−0.15^**^	−0.01	−0.02	0.13^**^	0.17^**^	0.01	−0.34^**^	(0.89)	
12. WDB-I	4.39	1.7	0.07	−0.29^**^	0.01	0.04	0.1^*^	−0.06	0.1	0.12^*^	−0.02	0.02	−0.01	(0.87)

*N* = 422. The square root of the AVE value of each latent variable is in parentheses. OBSE, organization-based self-esteem; LMX, leader–member exchange; WDB-O, organizational deviance behavior; WDB-I, interpersonal deviance behavior.

* *p* < 0.05;

** *p* < 0.01.

#### Content Validity

The KMO values of the latent variables attachment anxiety (0.93), attachment avoidance (0.94), LMX (0.94), OBSE (0.92), WDB-O (0.94), and WDB-I (0.94) were all greater than 0.8, and their scores on Bartlett’ s Test passed the significance test, showing that the questionnaire has high content validity.

#### Convergence Validity

The AVE values of the latent variables attachment anxiety (0.75), attachment avoidance (0.77), LMX (0.77), OBSE (0.68), WDB-O (0.79), and WDB-I (0.75) were all greater than 0.5, indicating that the latent variables in the questionnaire had high convergence validity. And the CR of the latent variables attachment anxiety (0.95), attachment avoidance (0.95), LMX (0.94), OBSE (0.92), WDB-O (0.96), and WDB-I (0.94) were all greater than 0.8, indicating that the reliability of the questionnaire is at a high level.

#### Discriminant Validity

As seen from Table 2, the square roots of the AVE values of attachment anxiety (0.86), attachment avoidance (0.88), LMX (0.87), OBSE (0.82), WDB-O (0.89), and WDB-I (0.87) were all greater than their Pearson correlation coefficient, showing that substitutability among the variables in the questionnaire is weak. Furthermore, we used confirmatory factor analysis to examine the validity of the differences among variables. The results showed that the 6-factor model was significantly better than other tissue models (*χ*^2^/df = 1.14, GFI = 0.94, AGFI = 0.92, NFI = 0.94, IFI = 0.99, TLI = 0.99, CFI = 0.99, RMSEA = 0.02, PGFI = 0.80, PCFI = 0.90), demonstrating that the variables had high discriminant validity.

### Common Method Bias Testing

Although the data used in this study were collected at different times, we still used the same sources of data to examine IAS, OBSE, LMX, and WDB. In accordance with the suggestion by Podsakoff et al. (2003), we used Harman’s single-factor test to check for common method bias. In the unrotated principal component analysis, a total of six factors were precipitated, and the factor with the highest variance explanation rate was 19.679% < 40%, so the model did not have a single common factor that could explain most of the variance.

Furthermore, we also adopted a stricter two-factor test to check for common method bias. The basic assumption of the two-factor model is that there is a global factor in the model that can explain the common variation of all measurement items. In addition, there are other local factors in the model that can explain the common variation of certain measurement items. We controlled for the influence of global factors to examine whether each local factor could fully explain the common variation of certain measurement items. We used SPSS 26.0 and Amos 23.0 to test the fitness of the six latent variable combination models of attachment anxiety, attachment avoidance, OBSE, LMX, WDB-O, and WDB-I (GFI = 0.93, AGFI = 0.91, NFI = 0.96, IFI = 0.99, TLI = 0.99, CFI = 0.99, RMSEA = 0.02, RMR = 0.09). On this basis, we added a global factor to test model fit. The results (GFI = 0.93, AGFI = 0.91, NFI = 0.96, IFI = 0.99, TLI = 0.99, CFI = 0.99, RMSEA = 0.01, RMR = 0.08) showed that there was no significant improvement in the key fitness indicators (>0.1), and the RMSEA and SRMR were not significantly reduced (<0.05). In summary, common method bias did not appear to be a serious problem for this research.

### Hypothesis Testing

**Figure 2:**
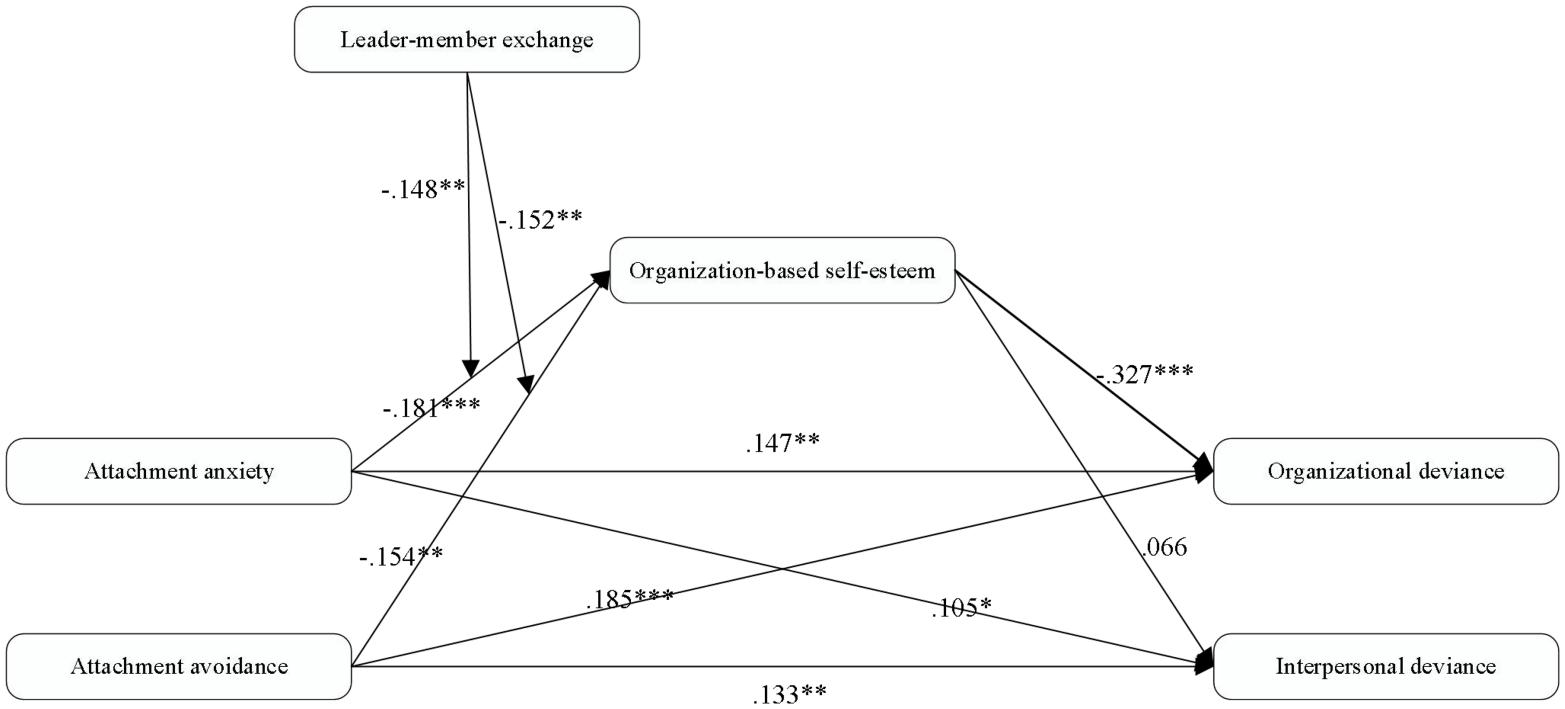
Standardized path coefficients for the direct and indirect effects of insecure attachment style (IAS) upon workplace deviation behavior (WDB) through organization-based self-esteem (OBSE) and moderated by leader–member exchange (LMX; *n* = 422). Participant age, gender, education, tenure, position, and tenure with supervisor are included as control variables. ^*^p < 0.05; ^**^p < 0.01; ^***^p < 0.001.

We conducted structural equation model to test the relationship between the two dimensions of IAS (attachment anxiety, attachment avoidance) and the two dimensions of WDB (WDB-O, WDB-I). As shown in Figure 2, the path coefficient of attachment anxiety to organizational deviance behavior was 0.147 (*p* < 0.01), and the path coefficient of attachment anxiety to interpersonal deviance behavior was 0.105 (*p* < 0.05). The path coefficient of attachment avoidance to organizational deviance behavior was 0.185 (*p* < 0.001), and the path coefficient of attachment avoidance to interpersonal deviance behavior was 0.133 (*p* < 0.01). Therefore, Hypothesis 1 is supported.

### Test of Mediation

Hypothesis 2 predicted that OBSE mediates the relationship between IAS and WDB. We conducted a bootstrapping analysis to test the statistical significance of the indirect effect. As the bias-corrected confidence interval did not include zero, the indirect effects of attachment anxiety [effect = 0.590, 95% CI = (0.03, 0.1)] and attachment avoidance [effect = 0.050, 95% CI = (0.02, 0.1)] on organizational deviance behavior through OBSE were statistically significant. However, the indirect effects of attachment anxiety [effect = −0.012, 95% CI = (−0.04, 0.1)] and attachment avoidance [effect = −0.010, 95% CI = (−0.04, 0.1)] on interpersonal deviance behavior through OBSE were not statistically significant, and the bias-corrected confidence interval included zero.

### Test of Moderation

We only tested the possibility that LMX may moderate the indirect effect of attachment anxiety and attachment avoidance on organizational deviance behavior. The interaction between attachment anxiety and LMX predicts OBSE (*b* = −0.148, *p* < 0.01), and the interaction between attachment avoidance and LMX predicts OBSE (*b* = −0.152, *p* < 0.01), both results were significant.

**Figure 3:**
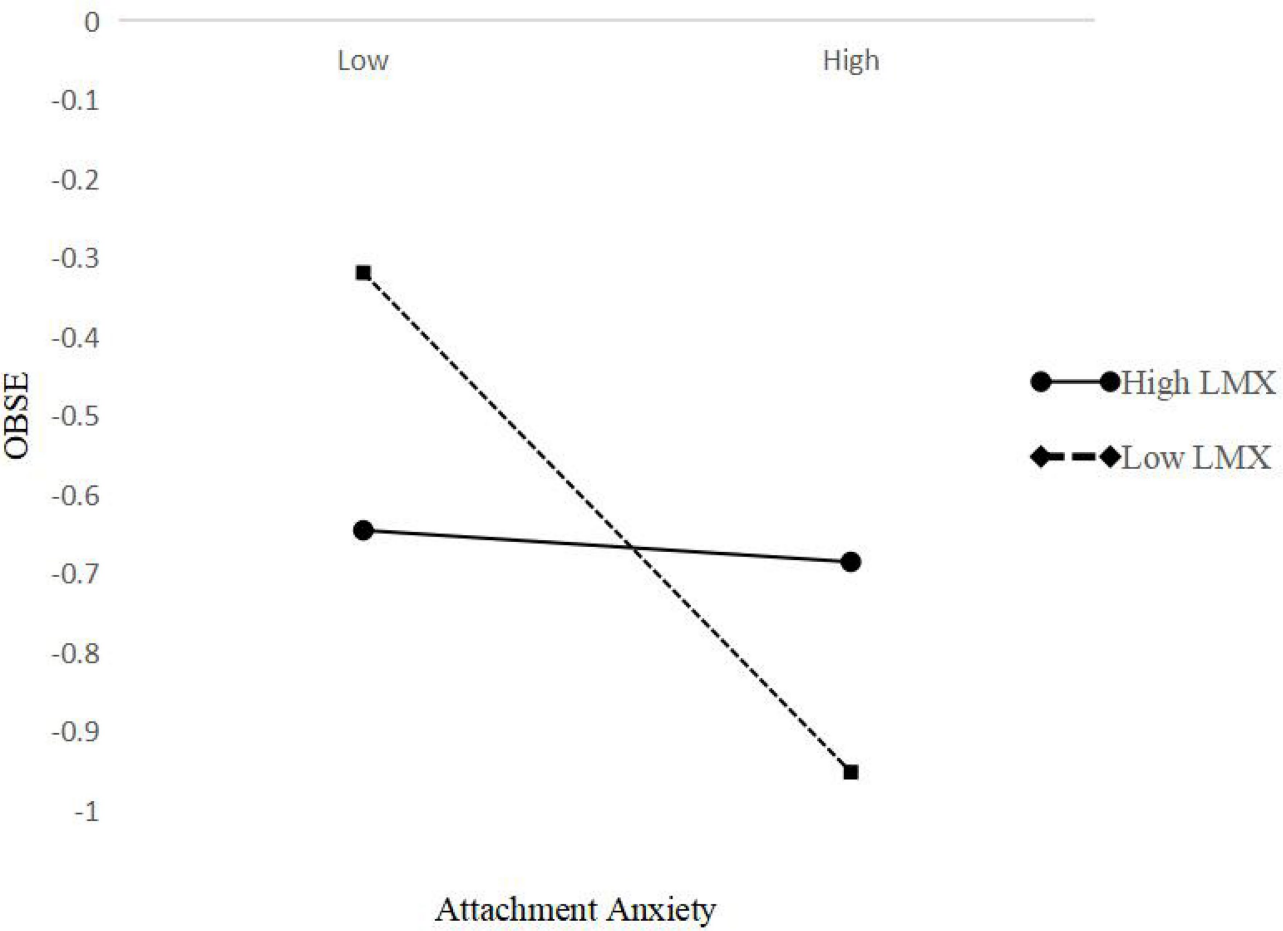
Interactive effects of attachment anxiety and leader–member exchange (LMX) on organization-based self-esteem (OBSE).

**Figure 4:**
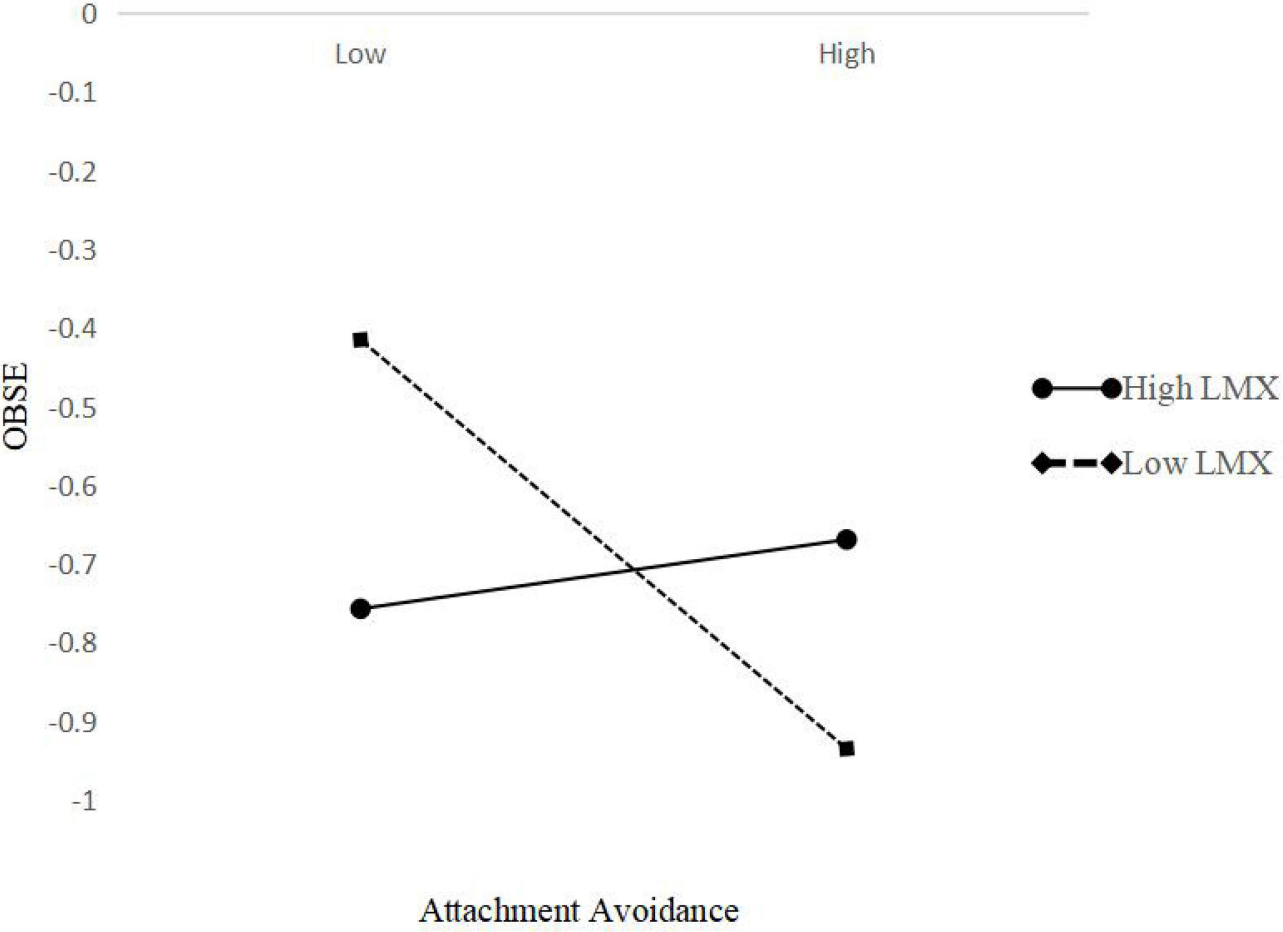
Interactive effects of attachment avoidance and leader–member exchange (LMX) on organization-based self-esteem (OBSE).

To present the moderating effect more intuitively, we used the program developed by Aiken and West (1991) to calculate the slope, and we also used standard deviations above and below the LMX average to plot the interaction. Figures 3, 4 show that the moderation effect was consistent with H3. Specifically, attachment anxiety and attachment avoidance were less negatively related to OBSE when LMX was higher.

Furthermore, we used the bootstrapping procedures developed by Edwards and Lambert (2007) to further test moderated mediation effect. As shown in Table 3, LMX moderated the indirect effects of both attachment anxiety [effect = 0.15, 95% CI = (0.08, 0.24)] and attachment avoidance [effect = 0.04, 95% CI = (0.04, 0.20)] on organizational deviance behavior *via* OBSE. Specifically, the indirect effects of attachment anxiety and attachment avoidance on organizational deviance behavior were weaker when LMX was higher.

**Table 3:** Results of the moderated mediation effect.

Moderator variable	Attachment anxiety→OBSE→WDB-O			Attachment avoidance→OBSE→WDB-O		
	Indirect effects	SE	95% CI (BCB)	Indirect effects	SE	95% CI (BCB)
High LMX (+1SD)	0.15	0.04	[0.08, 0.24]	0.11	0.04	[0.04, 0.20]
Low LMX (−1SD)	−0.01	0.02	[−0.04, 0.03]	0.01	0.02	[−0.04, 0.04]
Differences	0.16	0.05	[0.08, 0.26]	0.12	0 0.05	[0.03, 0.21]

*N* = 422. SE, standard error; CI, confidence interval; BCB, Bias-Corrected bootstrap.

## Discussion

We explored the linking mechanism underlying IAS and WDB in the context of a sample of 422 Chinese employees. Based on attachment theory, we focused on the mediating role of OBSE and the moderating role of LMX. Using a time-lagged research design, we concluded that attachment anxiety and attachment avoidance predict organizational deviance behavior and interpersonal deviance behavior. In past studies, organization (interpersonal injustice, organizational power), leader (leader mistreatment, abusive supervision) and colleagues (workplace ostracism) have proven to be external factors inducing WDB, while B5, HEXACO and personality have proven to be endogenous factors inducing WDB. Our results are consistent with the finding by Little et al. (2011) that counter-dependent attachment style positively predicts deviance. However, the positive correlation between attachment anxiety and WDB was not confirmed in Little’s study, which is inconsistent with our study. This result may be due to the fact that the data used by those authors to study deviant behavior were not self-reported but reported by supervisors, since not all deviant behavior data are observed by supervisors. Another reason for this result may be that the deviant behaviors observed by supervisors are more relevant to the organization, and the study by those authors does not further distinguish between deviant behavior dimensions.

Additionally, previous studies have regarded vigor, moral disengagement and feelings of violation as mediators of individual WDB. Although Little et al. (2011) proved the mediating role of vigor between IAS and WDB, we introduced organizational self-esteem into our model based on attachment theory and proved the mediating role of OBSE between IAS and WDB-O. Moreover, LMX weakened the negative effect of attachment anxiety and attachment avoidance on OBSE and further moderated the indirect effect of attachment anxiety and attachment avoidance on WDB-O *via* OBSE.

### Theoretical Implications

First, this study explored the relationship between IAS and WDB in the context of the COVID-19 epidemic, which provided a new theoretical framework for further study of WDB. Previous studies have discussed WDB from the perspective of internal or external causes, which provides sufficient support for explaining the antecedents of WDB. However, these studies have lacked explanations regarding why employees’ WDB increased during the epidemic. Based on attachment theory, we validated the relationship between IAS and WDB during the epidemic. Our study provides a new theoretical framework to explain WDB behavior during the epidemic and enriches the research concerning the antecedents of WDB.

Second, based on attachment theory, our research introduces OBSE into this model, which enriches the research concerning IAS and the internal mechanism of WDB. Previous studies have considered a sense of moral disengagement (Loi et al., 2020) and a feeling of violation as mediators of individual WDB. Based on the context of employee insecurity caused by the coronavirus outbreak and the internal working model of attachment theory, we selected OBSE as a mediator. Our study confirms the mediating role of OBSE in the relationship between IAS and WDB-O. This finding explains the increase in WDB during COVID-19 as the result of a decrease in OBSE among employees with IAS.

Third, from the perspective of the security needs of IAS, we take LMX as a moderating variable to expand the boundary conditions for alleviating WDB. Most scholars have viewed resignation intention and employee competency uncertainty as factors that can strengthen or alleviate WDB from the perspectives of power/dependence theory and self-uncertainty theory (Tepper et al., 2009; Mayer et al., 2012). In accordance with attachment theory, we explore the role of LMX in improving the OBSE of insecure adherents and reducing WDB to provide a new perspective on boundary research pertaining to WDB.

### Managerial Implications

Our research found a positive relationship between IAS and WDB, which means that the higher the degree of attachment anxiety and attachment avoidance is, the more likely that WDB will occur. Although attachment style is formed in early childhood, the internal working model of individual attachment is a continuously integrated structure, and supervisors can use a variety of methods to prevent or reduce WDB. First, supervisors can reduce employees’ anxiety or avoidance through psychological interventions. Companies can regularly carry out psychological training or open a psychological counseling office and use one-on-one psychological interviews to assist employees with serious insecure attachment. Studies have shown that therapeutic contact between employees and psychologists can help reduce insecure attachment (Hardy and Barkham, 1994).

Second, supervisors can increase employees’ OBSE through timely incentives. Studies have shown that employees with IAS are more sensitive to their relationships with their supervisors and colleagues because they did not receive sufficient and affirmative care from caregivers during childhood. Such employees become reliant on people’s perceptions of themselves (attachment anxiety) or completely refuse to contact other people to avoid harm (attachment avoidance) and thus feel lower OBSE. Therefore, they need more affirmation and encouragement than do ordinary employees. We suggest that supervisors pay more attention to such employees and promptly provide small verbal or material incentives when they achieve small goals in their work. Long-term affirmation and encouragement will cause these employees to become more confident and independent at work and to become more trustworthy within the organization, thereby increasing their sense of self-worth in the context of the organization.

Third, supervisors can establish high-quality LMX relationships with employees. Our research found that given the moderating effect of high-quality LMX, the downward trend in OBSE can be mitigated. Therefore, as objects of attachment in the workplace, supervisors can consciously establish high-quality LMX relationships with employees with IAS. In this process, supervisors can respond to the needs of those employees for protection and care and help employees modify their internal working models by exhibiting secure attachment behavior (Little et al., 2011).

### Limitations and Future Research Directions

Although this research was conducted as objectively as possible, it still faces a number of limitations. First, our data were taken from the same source and were all obtained through self-reporting. Although we adopted two-stage data collection methods to reduce the possibility of bias, and although the single-factor test and two-factor test showed that such bias was not a serious problem in this study, workplace deviance behavior itself has negative effects on employees and may affect their own benefits. Therefore, respondents may not have provided completely honest answers due to social desirability bias. Therefore, it is suggested that a more objective evaluation method could be used in the future.

Second, this study uses attachment theory to explain how IAS predicts WDB, which provides a new perspective for future research. However, there may be other theoretical frameworks to explain the intrinsic mechanisms underlying this context. For example, relational demand matching theory and self-uncertainty theory can explain the underlying mechanism linking different personality traits to work-related results (Ehrhardt and Ragins, 2016). In addition, we encourage the use of longitudinal data, such as by allowing employees to report their OBSE and organizational deviance behavior on a daily basis, as well as the use of experimental methods to obtain further causal inferences.

We selected LMX as a moderator at the organizational level according to the external-others model of the internal working model of attachment, and this study examined the ways in which LMX of different quality levels affects the relationship between IAS and OBSE. We look forward to exploring other moderators that alleviate or exacerbate the negative effect of IAS to enrich this research in the future, such as by examining employment opportunities in the external environment of organizations or personal psychological states of employees, such as trust.

Finally, the respondents in this study all had a common cultural background (a collectivist cultural background). In this context, employees’ emphasis on interactive relationships may have been strengthened. Arrindell (2003) found that individuals in China give more attention to the quality of interpersonal relationships. Therefore, we suggest that more research be carried out in different cultural contexts in the future.

## Conclusion

We focus on WDB during the COVID-19 pandemic and propose a new perspective of attachment theory concerning IAS to predict organizational deviance behavior. According to the internal working model of attachment theory, we take OBSE and LMX as the mediator and the moderator to explore the relationship between IAS and organizational deviance behavior. Our study expands the ability to predict and interpret organizational deviance behavior among employees, as our questions focus on the reasons why WDB behavior among employees increased during the pandemic. Simultaneously, our study provides a new theoretical framework for future research concerning employee negative behavior. The results of our study may also be applicable to the task of explaining negative employee behavior in an unsettled organizational climate. The reason for this possibility is that our research focuses on employees who are sensitive to the organizational atmosphere and confirms the positive relationship between these employees and WDB and the mechanism underlying that relationship; this research also addresses ways of mitigating the WDB of these employees. We also expect supervisors to be able to understand the psychological activities of employees with IAS by considering our research, and we provide theoretical references for formulating corresponding solutions.

## Data Availability Statement

The raw data supporting the conclusions of this article will be made available by the authors, without undue reservation.

## Author Contributions

WY collected data and wrote the first draft. HZ, ZL, XS, and JL provided valuable suggestions for the first draft. All authors contributed to the article and approved the submitted version.

## Funding

This research was supported by the Key Project of Beijing Social Science Fund (Project ID: 18GLA003).

## Conflict of Interest

The authors declare that the research was conducted in the absence of any commercial or financial relationships that could be construed as a potential conflict of interest.

## Publisher’s Note

All claims expressed in this article are solely those of the authors and do not necessarily represent those of their affiliated organizations, or those of the publisher, the editors and the reviewers. Any product that may be evaluated in this article, or claim that may be made by its manufacturer, is not guaranteed or endorsed by the publisher.

## Appendix

The measure items used in this study.

**Table:**

**Insecure attachment style**
Attachment avoidance
I want to get close to my partner, but I keep pulling back. I am nervous when partners get too close to me. I try to avoid getting too close to my partner. I usually discuss my problems and concerns with my partner. It helps to turn to my romantic partner in times of need. I turn to my partner for many things, including comfort and reassurance.
Attachment anxiety
I worry that romantic partners will not care about me as much as I care about them. My desire to be very close sometimes scares people away. I need a lot of reassurance that I am loved by my partner. I do not often worry about being abandoned. I find that my partner(s) do not want to get as close as I would like. I get frustrated if romantic partners are not available when I need them.
**Organization-based self-esteem**
I count around here. I am taken seriously. I am important. I am trusted. There is faith in me. I can make a difference. I am valuable. I am helpful. I am efficient. I am cooperative.
**Leader–member exchange**
I usually know how satisfied my leader is with what I do. My leader well understand my job problems and needs. My leader well recognize my potential. Regardless of how much formal authority my leader has built into his/her position, he/she would use his/her power to help me solve problems in my work. Again, regardless of the amount of formal authority my leader has, he/she would “bail me out,” at his/her expense. I have enough confidence in my leader that I would defend and justify his/her decision if he/she were not present to do so. I have a good relationship with my leader?
**Workplace deviance behavior**
Organizational deviance behavior
Taken property from work without permission. Spent too much time fantasizing or daydreaming instead of working. Falsified a receipt to get reimbursed for more money than you spent on business expenses. Taken an additional or longer break than is acceptable at your workplace. Come in late to work without permission. Littered your work environment. Neglected to follow your boss’s instructions. Intentionally worked slower than you could have worked. Discussed confidential company information with an unauthorized person. Used an illegal drug or consumed alcohol on the job. Put little effort into your work. Dragged out work in order to get overtime.
Interpersonal deviance behavior
Made fun of someone at work. Said something hurtful to someone at work. Made an ethnic, religious, or racial remark at work. Cursed at someone at work. Played a mean prank on someone at work. Acted rudely toward someone at work. Publicly embarrassed someone at work.
